# School-level peer digital distraction, mathematics performance, creative thinking, and adolescent well-being across 80 education systems: global evidence from PISA 2022

**DOI:** 10.7189/jogh.16.04351

**Published:** 2026-11-06

**Authors:** Chien-Mei Sung, Chia-Huei Lin, Hsueh-Hsing Pan, Ke-Feng Lin, Yen-Chung Ho

**Affiliations:** 1 College of Nursing, National Defense Medical University, Taipei, Taiwan, R.O.C.; 2 Department of Nursing, Tri-Service General Hospital, National Defense Medical University, Taipei, Taiwan, R.O.C.; 3 Department of Medical Planning and Management, Tri-Service General Hospital, National Defense Medical University, Taipei, Taiwan, R.O.C.

**Keywords:** adolescent well-being, creative thinking, digital distraction, education, PISA, school policy

## Abstract

**Background:**

Digital-device debates in schools often focus on students’ own use or nominal phone bans. We examined whether school-level peer digital distraction, conceptualised as a shared classroom exposure, was associated with mathematics performance, creative thinking, and life satisfaction across PISA 2022 systems.

**Methods:**

In this cross-sectional observational study, we used public PISA 2022 student, school, and creative-thinking data. The analytic file included 613,744 students from 21,629 schools in 80 countries/economies, representing approximately 28.3 million 15-year-old students. Full adjusted complete-case samples included 77,216 students for mathematics, 65,222 for creative thinking, and 67,748 for life satisfaction. The exposure was a weighted leave-one-out school-level peer digital-distraction measure. Models used country/economy fixed effects, PISA final student weights, 80 Fay balanced repeated replication weights, and plausible-value pooling.

**Results:**

We found that higher school-level peer digital distraction was associated with lower mathematics performance (*β* = −34.28; standard error (SE) = 3.95; 95% confidence interval (CI) = −42.02, −26.54, *P* < 0.001) and lower creative-thinking performance (*β* = −3.81; SE = 0.53; 95% CI = −4.85, −2.76, *P* < 0.001). Per within-country standard deviation of the exposure, the adjusted associations were −11.31 points for mathematics and −1.19 points for creative thinking. Phone-ban interactions were not statistically significant. School-level peer digital distraction was not significantly associated with life satisfaction.

**Conclusions:**

Across a large global PISA 2022 file, school-level peer digital distraction was consistently associated with lower mathematics and creative-thinking performance in complete-case adjusted models. Findings are cross-sectional associations and should not be interpreted as causal evidence of educational harm or policy effectiveness.

Digital resources are embedded in adolescents’ school and social lives. PISA 2022 documented learning disruption, equity gaps, and marked cross-system variation in digital learning environments [1,2]. The Organisation for Economic Co-operation and Development (OECD) and United Nations Educational, Scientific and Cultural Organization note that technology can widen access to educational resources, but that its educational benefits depend on appropriate pedagogy and governance [3,4]. The relevant question is therefore not access to digital resources alone, but how these resources are used in classrooms. Critical scholarship treats education technology as a socio-technical and institutional issue [5,6]. Work on school phone bans similarly frames policy as a means to study addiction, distraction, and cyberbullying [7]. A PISA 2022 analysis highlights enforcement and classroom dynamics but examines restriction policies, not peer digital distraction as a school exposure [8].

Reviews and meta-analyses associate students’ own smartphone or mobile-phone use with poorer academic outcomes [9–11], but do not evaluate classmates’ second-hand exposure. Broader reviews show that digital-distraction findings vary by task, context, and measurement [12,13]. Treating all screen activity as one exposure is therefore unwarranted. A media-multitasking review reports heterogeneous academic consequences [14]. Observational studies link in-class multitasking with performance but mainly measure students’ own behaviour [15,16]. Related studies link social networking with performance [17] and cell-phone use with performance, anxiety, and life satisfaction [18]. Both remain vulnerable to self-selection and common-method bias.

A quasi-experimental study in English secondary schools found improved examination scores following mobile-phone bans [19]. A randomised trial in an introductory economics course at the United States Military Academy found higher final examination scores in classrooms that prohibited computers [20]. Their stronger designs evaluate rules or access, not peer distraction. Experiments show that instant messaging, off-task technology use, and divided attention can impair immediate learning [21–23]. Their controlled tasks represent short learning episodes. Other experiments link in-class phone use and text-message-induced task switching with poorer learning [24,25]. They test students’ own switching, not a school peer climate. Cognitive studies suggest that media multitasking, notifications, and smartphone presence can tax attention and working memory [26–28]. These mechanisms make spillover plausible but do not establish its magnitude. A cognitive review finds non-uniform associations across domains and users [29]. This literature motivates, but does not validate, a shared school exposure.

Sana *et al.* showed that nearby peers of laptop multitaskers learned less [30]. Other classroom studies describe altered norms, attention, and teacher responses [31,32]. We extend this work to a leave-one-out school exposure, mathematics and creative-thinking outcomes, phone-policy context, and PISA variance estimation across 80 systems. Furthermore, ecological perspectives frame classrooms as nested social systems [33,34]. This supports environmental aggregation while preserving the distinction between context and causation. Engagement research links attention, motivation, and cognitive engagement with learning [35,36]. Peer distraction is one possible feature of that environment, not a complete account of the factors influencing learning. Research on visible learning, affective neuroscience, and achievement-emotion connects learning with instructional and emotional conditions [37–39]. Attention is therefore partly contextual. Reviews on classroom management and school climate associate shared conditions with academic and behavioural outcomes [40–42]. They justify school-level description but do not solve selection or correlated-effects problems. Peer composition can pattern achievement [43,44], while socioeconomic gradients remain strong [45]. We therefore examine a contextual association, not an identified causal peer effect.

Research on creativity distinguishes performance levels and the roles of convergent and divergent thinking [46–48]. Creative thinking is therefore related to, but not interchangeable with, conventional achievement. Classroom creativity depends on environments that permit idea generation, evaluation, and improvement [49–51]. The surrounding attention environment may therefore matter. PISA 2022 provides a standardised cross-national creative-thinking assessment [52]. Its plausible values permit analysis beyond conventional achievement with formal PISA variance estimation.

Digital-reading evidence shows that medium effects vary by task and comprehension demand [53,54]. Device presence must therefore be distinguished from activity and context. Digital-divide research shows that access does not guarantee equivalent skills or use [55,56]. This matters when interpreting individual digital covariates. Large-scale adolescent studies find small or heterogeneous digital-technology associations with well-being [57–59]. We examined school peer distraction in relation to mathematics, creative thinking, and life satisfaction, with policy, socioeconomic, and cross-national sensitivity analyses.

## METHODS

Because this study is a secondary analysis of a large public international data repository, we followed JoGH’s Guidelines for Reporting Analyses of Big Data Repositories Open to the Public (GRABDROP) [60]. We reported the originality of the research question, related analyses in the same repository, multiple-testing approach, and use of artificial intelligence-assisted language editing in Table S1 in the **Online Supplementary Document**.

### Study design and data source

In this cross-sectional observational analysis, we used PISA 2022 public data. We used the student questionnaire file, school questionnaire file, and creative-thinking data file. PISA assesses 15-year-old students through standardised assessment frameworks and documented sampling procedures [61,62]. We used student final weights, replicate weights, and plausible values according to PISA secondary-analysis guidance [63,64]. The full corrected analytic file included 613,744 student records nested in 21,629 schools across 80 countries/economies, representing approximately 28.3 million students after applying PISA final student weights. The adjusted models estimate association and do not identify causal effects.

### Exposure

The primary exposure was weighted leave-one-out school-level peer digital distraction. It was derived from PISA item ST273Q07JA, which asked how often students are distracted by other students using digital resources. Responses were recoded so higher values indicated more frequent peer distraction (peer_distract_individual = 5 - ST273Q07JA). For each student, the school-level exposure was calculated as the weighted mean among other sampled students in the same school, excluding the index student’s own response. This construction reduces same-source overlap with the index student’s own report but remains a school aggregate of student perceptions rather than an independently observed measure of peer behaviour (Figure S1 in the **Online Supplementary Document**).

### Outcomes and covariates

The primary cognitive outcomes were PISA mathematics plausible values (PV1MATH through PV10MATH) and creative-thinking plausible values (PV1CRTH_NC through PV10CRTH_NC). We analysed life satisfaction, derived from ST016Q01NA on a 0-10 scale, as a secondary well-being outcome. Individual digital covariates included individual perceived peer distraction, own digital distraction in mathematics lessons, digital device open in class, in-school leisure digital-resource use, digital anxiety, and pressure to be online or respond in class. We included these covariates to adjust for correlated individual digital experiences, but they may also lie on, or be affected by, pathways linking school climate and outcomes; therefore, we did not interpret adjusted coefficients as causal mediation estimates. We derived school cell-phone ban status from school questionnaire item SC190Q02JA. We modelled country/economy using fixed effects. We used World Bank income groups and United Nations (UN) M49 regions only for heterogeneity analyses [65,66].

### Missing data and analytic samples

The formal adjusted models were estimated using listwise complete cases for the outcome, country/economy fixed effect, final student weight, school-level exposure, digital covariates, phone-policy variable, and interaction term. The PV1 complete-case samples were 77,216 students for mathematics, 65,222 for creative thinking, and 67,748 for life satisfaction, representing 3,071,053, 2,505,922, and 2,915,653 students, respectively, after applying final student weights. Missingness was driven mainly by student digital questionnaire items (Table S2 in the **Online Supplementary Document**). The life-satisfaction analysis was retained as secondary because of its complete-case restriction.

### Statistical analysis

All cognitive-outcome models incorporated PISA 2022 final student weights (W_FSTUWT), the 80 final student replicate weights, Fay balanced repeated replication with Fay factor 0.5, and all 10 plausible values. Estimates were obtained for each plausible value and replicate-weight combination; sampling variance was computed using Fay’s balanced repeated replication (BRR), and plausible-value uncertainty was pooled using Rubin’s rules. The primary adjusted model included country/economy fixed effects, school-level leave-one-out peer digital distraction, individual perceived peer distraction, own digital distraction in mathematics lessons, device open in class, in-school leisure digital-resource use, digital anxiety, pressure to be online/respond in class, school cell-phone ban status, and the school peer-distraction by phone-ban interaction. We used the same adjusted structure for life satisfaction, estimating the model once with the full weight and once with each replicate weight because life satisfaction is not a plausible-value outcome.

We performed all formal cognitive-outcome analyses using SPSS Statistics, version 24 (IBM Corp., Armonk, New York, USA).

### Diagnostic and multiplicity approach

To address measurement and interpretability, we calculated the distribution of the school-level exposure, the correlation between individual perceived peer distraction and the leave-one-out school mean, unweighted intraclass correlation coefficients (ICCs) for aggregation of the single item, with ICC(1) describing the proportion of item variance between schools and ICC(2) the reliability of the school mean at the unbalanced average cluster size, and variance inflation factors among the main digital covariates. Confirmatory interpretation focused on the prespecified school-level peer-distraction association for mathematics and creative thinking. Sensitivity, heterogeneity, and contextual stratification analyses were treated as robustness or exploratory analyses. We reported *P*-values to quantify compatibility with the models, but did not use them to support additional causal or policy-effect claims.

### Sensitivity, secondary, and heterogeneity analyses

Prespecified robustness analyses examined minimum valid peer-count thresholds within schools, adjustment-set complexity, OECD *vs.* non-OECD/partner restrictions, creative-thinking participating systems, phone-policy complete-case restriction, exclusion of countries/economies with extreme peer-distraction burden, leave-one-country/economy-out influence screening, and alternative school-level exposure definitions. Formal heterogeneity analyses examined OECD status, socioeconomic status using the index of economic, social and cultural status (ESCS), World Bank income group, and UN M49 broad geographic region. Contextual stratification and demographic/socioeconomic status (SES)-adjusted analyses were retained as secondary screening diagnostics unless explicitly labelled as full Fay’s BRR analyses. Detailed robustness estimates are reported in Tables S4–10, core and extended heterogeneity results in Tables S11–14, and the secondary life-satisfaction model in Table S15 in the **Online Supplementary Document**.

## RESULTS

### Analytic sample and global coverage

The full corrected analytic file included 613,744 students from 21,629 schools in 80 countries/economies, representing approximately 28.3 million 15-year-old students after applying PISA final student weights. The weighted population comprised 13.9 million students in OECD systems and 14.4 million in non-OECD or partner systems. School-reported cell-phone bans applied to 36.5% of the weighted student population: 32.2% in OECD systems and 40.5% in non-OECD or partner systems. Mean school-level peer distraction was also lower in OECD systems (1.85) than in non-OECD or partner systems (2.04), indicating descriptive differences in both nominal policy prevalence and reported peer-distraction climate (**Figure 1**, **Table 1**). The unweighted mean phone-ban prevalence across the 80 country/economy profiles was 40.7%; this summary gives each system equal weight and therefore differs from the pooled student-weighted prevalence (Table S3 and Figure S3 in the **Online Supplementary Document**). Complete-case samples were 77,216 for mathematics, 65,222 for creative thinking, and 67,748 for life satisfaction, reflecting substantial restriction because of missing digital-questionnaire and school phone-policy items (Table S2 in the **Online Supplementary Document**).

**Figure 1:**
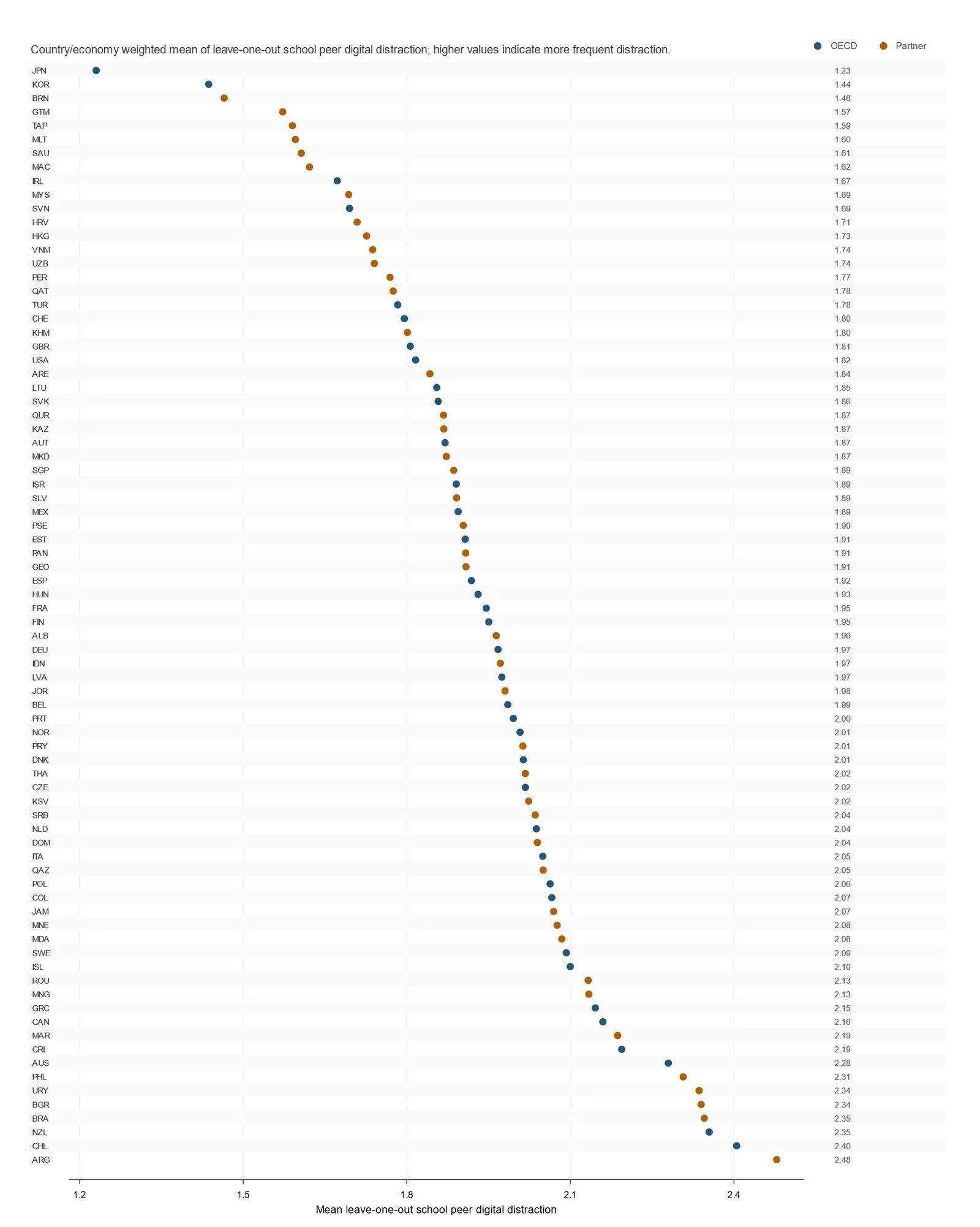
Global distribution of country/economy mean school-level peer digital distraction.

**Table 1:** Sample characteristics and cross-national coverage of the corrected PISA 2022 analytic data set

Characteristics	Overall	OECD systems	Non-OECD/partner systems
Countries/economies, n	80	37	43
Schools, n	21,629	11,108	10,521
Students, unweighted n	613,744	295,157	318,587
Weighted student population	28,255,206	13,891,949	14,363,257
Female, weighted %	50.4	50.2	50.7
Immigrant background, weighted %	8.1	14.8	2.2
Grade repetition, weighted %	12.8	10.1	15.1
ESCS, weighted mean	−0.69	−0.19	-1.15
Individual perceived peer distraction, weighted mean	1.93	1.84	2.01
Leave-one-out school peer distraction, weighted mean	1.95	1.85	2.04
Valid peer reports per school exposure, weighted mean	21.2	20.3	22.0
Own digital distraction in mathematics lessons, weighted mean	2.02	1.98	2.05
Digital device open in class, weighted mean	2.70	2.63	2.76
Leisure digital-resource use at school, weighted mean	2.27	2.15	2.36
Digital anxiety, weighted mean	1.71	1.71	1.71
Pressure to be online/respond in class, weighted mean	1.54	1.44	1.62
School cell-phone ban, weighted %	36.5	32.2	40.5
Creative-thinking PV available, weighted %	69.2	51.5	86.2
Mathematics plausible-value mean	424.3	465.7	384.3
Creative-thinking plausible-value mean	24.5	32.0	20.2
Life satisfaction, weighted mean	6.90	6.56	7.12

ESCS – index of economic, social and cultural status, OECD – Organisation for Economic Co-operation and Development, PV – plausible value

### Primary adjusted associations

In formal BRR/plausible-value models using 10 plausible values and 80 replicate weights, school-level leave-one-out peer digital distraction was negatively associated with both mathematics and creative-thinking performance (**Figure 2**, **Table 2**). For mathematics, a one-unit increase in school peer digital distraction was associated with a 34.28-point lower mathematics score (standard error (SE) = 3.95; 95% confidence interval (CI) = −42.02, −26.54, *P* < 0.001). For creative thinking, the corresponding association was a 3.81-point lower creative-thinking score (SE = 0.53; 95% CI = −4.85, −2.76, *P* < 0.001). These estimates were adjusted for country/economy fixed effects, individual perceived peer distraction, own digital distraction in mathematics lessons, device open in class, in-school leisure digital-resource use, digital anxiety, pressure to be online/respond in class, school cell-phone ban status, and the school peer-distraction by phone-ban interaction.

**Figure 2:**
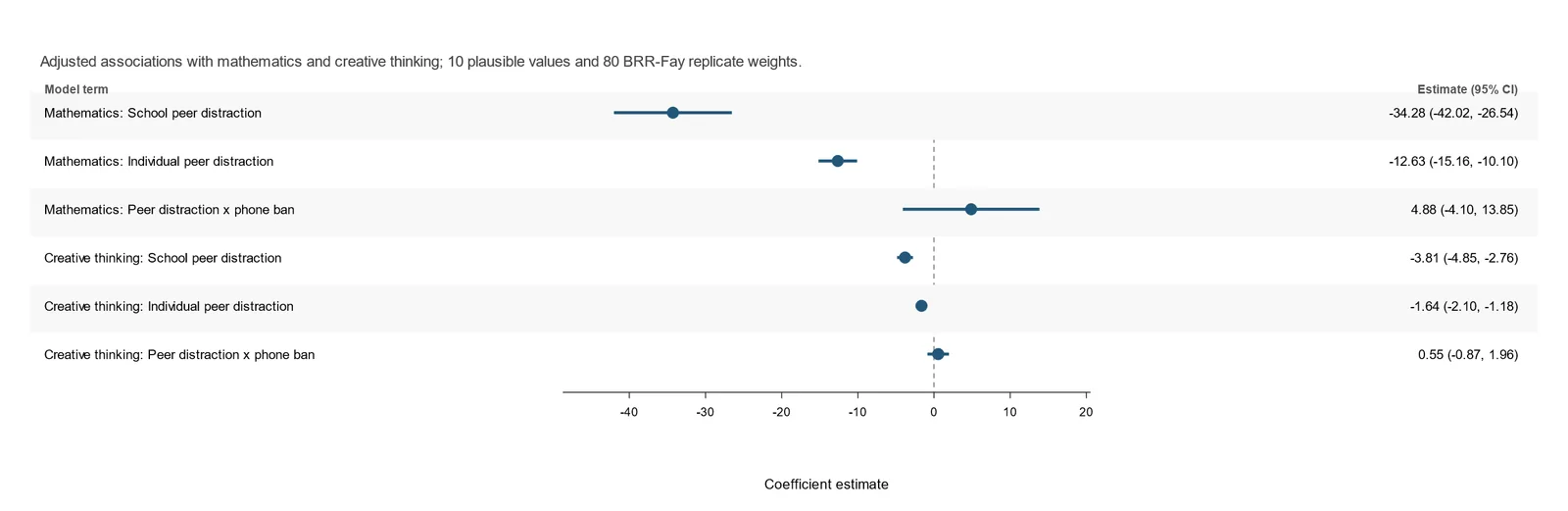
Primary Fay’s balanced repeated replication/plausible-value coefficient plot for mathematics and creative thinking.

**Table 2:** Full adjusted associations of school-level and individual peer digital distraction with mathematics and creative-thinking performance

Outcome	Estimate	SE (95% CI)	*P*-value	PV	Replicates
Creative thinking					
*Digital device open in class*	0.73	0.08 (0.56, 0.89)	<0.001	10	80
*Digital anxiety*	0.77	0.13 (0.51, 1.03)	<0.001	10	80
*Own digital distraction in mathematics lessons*	0.69	0.23 (0.25, 1.14)	0.002	10	80
*Individual perceived peer distraction*	−1.64	0.23 (−2.10, −1.18)	<0.001	10	80
*Leave-one-out school peer distraction*	−3.81	0.53 (−4.85, −2.76)	<0.001	10	80
*School peer distraction x phone ban*	0.55	0.72 (−0.87, 1.96)	0.448	10	80
*School cell-phone ban*	−2.19	1.48 (−5.08, 0.70)	0.139	10	80
*Pressure to be online/respond in class*	−0.85	0.15 (−1.14, −0.55)	<0.001	10	80
*Leisure digital-resource use at school*	−0.06	0.10 (−0.26, 0.13)	0.515	10	80
Mathematics					
*Digital device open in class*	4.28	0.50 (3.31, 5.25)	<0.001	10	80
*Digital anxiety*	2.70	0.65 (1.42, 3.98)	<0.001	10	80
*Own digital distraction in mathematics lessons*	4.87	1.36 (2.21, 7.53)	<0.001	10	80
*Individual perceived peer distraction*	−12.63	1.29 (−15.16, −10.10)	<0.001	10	80
*Leave-one-out school peer distraction*	−34.28	3.95 (−42.02, −26.54)	<0.001	10	80
*School peer distraction x phone ban*	4.88	4.58 (−4.10, 13.85)	0.287	10	80
*School cell-phone ban*	−18.66	9.19 (−36.67, −0.65)	0.042	10	80
*Pressure to be online/respond in class*	−6.50	0.87 (−8.19, −4.80)	<0.001	10	80
*Leisure digital-resource use at school*	−1.79	0.50 (−2.78, −0.80)	<0.001	10	80

CI – confidence interval, PV – plausible value, SE – standard error

### School cell-phone policy and other digital covariates

The interaction between school-level peer digital distraction and school cell-phone ban status was not statistically significant for mathematics (*β* = 4.88; SE = 4.58; 95% CI = −4.10, 13.85, *P* = 0.287) or creative thinking (*β* = 0.55; SE = 0.72; 95% CI = −0.87, 1.96, *P* = 0.448). The main effect of school cell-phone ban status was negative for mathematics (*β* = −18.66; SE = 9.19; 95% CI = −36.67, −0.65, *P* = 0.042) and not statistically significant for creative thinking (*β* = −2.19; SE = 1.48; 95% CI = −5.08, 0.70, *P* = 0.139). Pressure to be online/respond in class was negatively associated with both outcomes: mathematics (*β* = −6.50; SE = 0.87; 95% CI = −8.19, −4.80, *P* < 0.001) and creative thinking (*β* = −0.85; SE = 0.15; 95% CI = −1.14, −0.55, *P* < 0.001) (**Table 2**).

### Measurement, missing-data, and collinearity diagnostics

The weighted mean of the school-level leave-one-out exposure was 1.951 (weighted standard deviation (SD) = 0.439), so a one-response-category contrast corresponded to approximately 2.28 weighted SDs on the observed student-weighted distribution. Alternative exposure analyses therefore also reported within-country SD-scaled estimates: mathematics *β* = −11.31 (95% CI = −14.07, −8.55, *P* < 0.001) and creative thinking *β* = −1.19 (95% CI = −1.58, −0.80, *P* < 0.001). The individual peer-distraction item and leave-one-out school mean were correlated but not redundant (weighted Pearson *r* = 0.303; weighted Spearman’s ρ = 0.315). The single-item school aggregation showed ICC(1) = 0.123 and ICC(2) = 0.726 at the unbalanced average cluster size. Variance inflation factors (VIFs) among the main digital covariates were not extreme (maximum VIF = 2.51), but the counterintuitive positive adjusted coefficients remain a warning against interpreting the full model as a causal decomposition (Table S2 in the **Online Supplementary Document**).

### Robustness and heterogeneity

Across adjustment specifications, school peer-distraction coefficients ranged from −38.53 to −31.13 for mathematics and from −4.57 to −3.73 for creative thinking (all *P* < 0.001), indicating that the negative associations did not depend on one adjustment set. When at least 15 valid peer reports were required, the coefficients were −44.69 for mathematics and −5.06 for creative thinking (both *P* < 0.001). Leave-one-country/economy-out screening yielded ranges of −37.78 to −31.01 and −4.52 to −3.34, respectively, indicating that no single system reversed the direction of either point estimate; these were final-weight screening estimates without BRR-based inference (**Figure 3**, **Table 3**, Tables S4, S5 and S9 in the **Online Supplementary Document**). In formal heterogeneity models, the non-OECD-minus-OECD slope difference was 23.20 mathematics points (95% CI = 11.13, 35.27, *P* < 0.001), indicating a less negative mathematics association in non-OECD systems. The corresponding creative-thinking difference was 1.49 points (95% CI = −0.23, 3.21, *P* = 0.089), providing less clear evidence of a difference. The peer-distraction-by-ESCS interaction was −6.24 for mathematics (95% CI = −9.57, −2.92, *P* < 0.001) and −0.34 for creative thinking (95% CI = −0.88, 0.20, *P* = 0.216); evidence of socioeconomic moderation was therefore clearer for mathematics, while the creative-thinking interval included zero (Table S11 and Figure S2 in the **Online Supplementary Document**).

**Figure 3:**
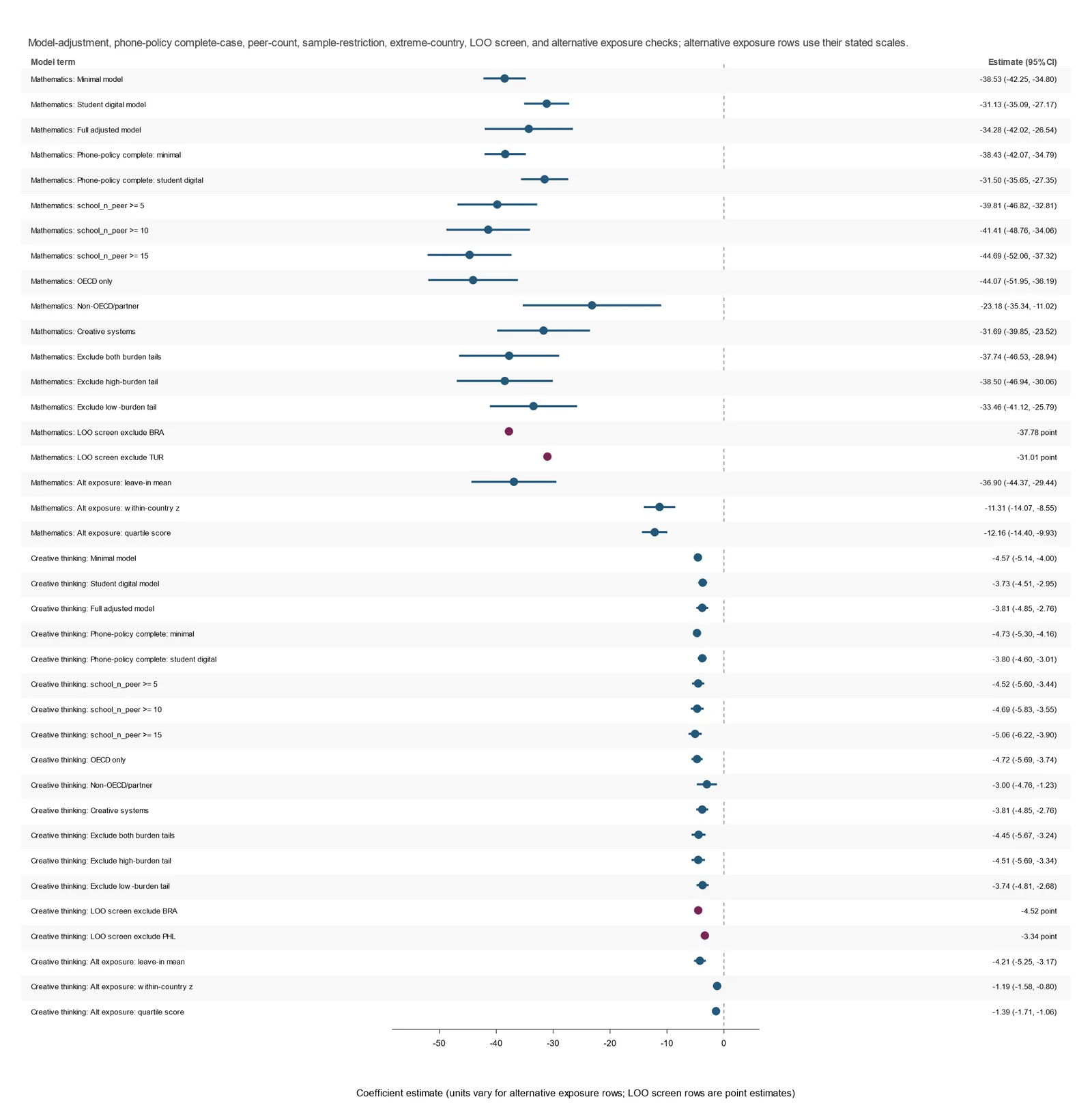
Robustness grid showing coefficient stability across sensitivity and influence analyses.

**Table 3:** Robustness, heterogeneity, and secondary-outcome summary

Evidence domain	Analysis summarised	Mathematics summary	Creative-thinking summary	Interpretation
Primary full adjusted model	Country/economy fixed effects; individual perceived peer distraction; own digital distraction; device open in class; leisure digital-resource use at school; digital anxiety; pressure to respond; school phone-ban status; peer distraction x phone ban	School peer distraction *β* = −34.28, *P* < 0.001	School peer distraction *β* = −3.81, *P* < 0.001	Primary school peer-distraction association was negative for both cognitive outcomes.
Model-adjustment sensitivity	Minimal country/economy fixed-effects, student digital covariate, and fully adjusted models	B ranged from −38.53 to −31.13; all *P* < 0.001	B ranged from −4.57 to −3.73; all *P* < 0.001	Association did not depend on a single covariate specification.
Minimum school peer-count thresholds	Full sample and schools with school_n_peer ≥5, ≥10, and ≥15	B ranged from −44.69 to −34.28; all *P* < 0.001	B ranged from −5.06 to −3.81; all *P* < 0.001	Association persisted when school peer exposure required more valid peer reports.
Sample restrictions	Full sample, OECD systems, non-OECD/partner systems, and creative-thinking participating systems	B ranged from −44.07 to −23.18; all *P* < 0.001	B ranged from −4.72 to −3.00; all *P* < 0.001	Association was not limited to one broad country/economy group.
Phone-policy complete-case sensitivity	Minimal and student digital models restricted to non-missing school phone-policy status	Estimates changed minimally; all *P* < 0.001	Estimates changed minimally; all *P* < 0.001	Missing school phone-policy status did not explain the primary association.
Extreme peer-burden country/economy exclusions	Excluded lowest and highest 5% peer-burden systems, highest 5% only, or lowest 5% only	*β* ranged from −38.50 to −33.46; all *P* < 0.001	*β* ranged from −4.51 to −3.74; all *P* < 0.001	Findings were not driven by a small number of unusually low- or high-distraction systems.
Alternative exposure definitions	Leave-in school mean, within-country standardised exposure, and quartile-score exposure	All alternative exposure estimates were negative and *P* < 0.001	All alternative exposure estimates were negative and *P* < 0.001	Finding was not an artefact of leave-one-out scaling or one exposure metric.
Phone-policy moderation	Peer distraction x premises-wide school cell-phone ban in primary and alternative exposure models	Primary interaction *β* = 4.88, *P* = 0.287	Primary interaction *β* = 0.55, *P* = 0.448	Nominal phone-ban status did not significantly moderate the peer-distraction association.
Core heterogeneity	OECD interaction and ESCS interaction	Stronger negative slope in OECD systems; ESCS interaction negative	OECD difference not statistically significant at 0.05; ESCS interaction not statistically significant	Heterogeneity was present but context-specific; subgroup findings should be interpreted carefully.
Secondary well-being outcome	Life satisfaction (ST016Q01NA) with final student weights and 80 Fay’s BRR replicate weights	NA	NA	School peer distraction was not statistically significant for life satisfaction, whereas individual peer distraction, own distraction, and digital anxiety were negative.

BRR – balanced repeated replication, ESCS – index of economic, social and cultural status, NA – not applicable, OECD – Organisation for Economic Co-operation and Development

### Secondary well-being outcome

We analysed life satisfaction in a complete-case model using the same digital covariates, school phone-policy term, phone-ban interaction, and country/economy fixed effects used in the cognitive-outcome models. The complete-case unweighted number was 67,748, representing 2,915,653 students after applying final student weights. School-level peer digital distraction was not significantly associated with life satisfaction (*β* = 0.129; SE = 0.082; 95% CI = −0.032, 0.291, *P* = 0.117). Individual perceived peer distraction (*β* = −0.102; SE = 0.037; 95% CI = −0.173, −0.030, *P* = 0.005) and own digital distraction in mathematics lessons (*β* = −0.080; SE = 0.036; 95% CI = −0.151, −0.009, *P* = 0.027) were negatively associated with life satisfaction (**Table 3**; Table S15 in the **Online Supplementary Document**).

## DISCUSSION

This cross-national PISA 2022 study examined school-level peer digital distraction as a shared learning-environment exposure. In complete-case models, students in schools with higher leave-one-out peer digital distraction had lower mathematics and creative-thinking performance after adjustment for country/economy fixed effects, individual digital covariates, and nominal phone-policy status. The pattern was stable across multiple robustness analyses, but it remains a cross-sectional association.

The direction of this association is consistent with meta-analytic and review evidence linking students’ own smartphone use, mobile-phone use, and media multitasking with poorer academic outcomes [9–11]. This literature chiefly concerns own behaviour, not shared exposure.

The result also accords qualitatively with classroom experiments in which technology-related task switching impaired immediate learning [21,23,25]. These tasks are short episodes, not school climates.

Cognition reviews report non-uniform associations [29]. The closest prior test of second-hand exposure is Sana *et al.*, who found that students seated in view of laptop multitaskers performed worse on lecture comprehension [30]. Our study extends that mechanism from a simulated university lecture to a school-level adolescent exposure across 80 systems, but it lacks experimental control. The convergence is directional, not an effect-size replication, because age, exposure, time scale, and outcome differ.

School-climate research offers another comparison. Reviews link shared classroom and school conditions with academic and behavioural outcomes [40–42]. This supports school-level description, not causal validation of the aggregated item.

Peer-effect studies likewise show that composition can predict achievement through social and selection processes [43,44]. Our negative school-level slope is compatible with a contextual interpretation, but it does not identify a contextual peer effect. Country/economy fixed effects remove between-system differences, and leave-one-out construction removes the index student's own response from the school mean; neither addresses within-country sorting, teacher quality, discipline, tracking, motivation composition, leadership, or digital-integration pedagogy. These correlated school factors could explain some or all of the association.

Magnitude requires a common scale. A one-response-category contrast was approximately 2.28 weighted SDs of the observed school exposure. Per within-country SD, the adjusted differences were −11.31 mathematics points and −1.19 creative-thinking points. These estimates cannot be compared directly with standardised meta-analytic effects for individual phone use [9,10] or with Sana *et al.*’s lecture-comprehension experiment [30].

Both exposure-SD contrasts were smaller than the ESCS benchmark, consistent with evidence that socioeconomic gradients remain major correlates of achievement [45]. Outcomes remain in PISA points, so the exposure-SD contrast is interpretable. Because the BRR fixed-effects models are not multilevel variance-components models, they do not estimate a share of between-school variance explained. We therefore interpret the estimates as nontrivial descriptive contrasts, not quantified learning loss.

Aggregation diagnostics do not settle measurement validity. The item showed ICC(1) = 0.123, ICC(2) = 0.726 at the unbalanced average cluster size, and school-level reliability interquartile range = 0.628, 0.779; its correlation with the individual report was moderate (weighted Pearson r = 0.303; Spearman rho = 0.315). Methodological work cautions that observed group means are often treated as perfectly reliable even though finite cluster sizes can distort group-level and contextual estimates [67]. Here, ICC(2) supports reproducibility of the aggregate, not content validity. The exposure remains one subjective item and may reflect response norms, school reporting culture, or performance-attributional bias. Classical measurement error would usually attenuate an otherwise unconfounded association; correlated reporting error or residual confounding could inflate or create it.

The adjusted covariate pattern warrants similar restraint. Device open in class, digital anxiety, and own digital distraction had positive coefficients in the full model. A maximum VIF of 2.51 argues against severe linear multicollinearity, but it does not exclude suppression, differential access, response-style differences, or conditioning on mediators or colliders. Digital-divide research also shows that access, skill, and type of use are not interchangeable [55,56]. More generally, adjustment for an intermediate variable can bias a total-effect estimate rather than purify it [68]. The minimal-to-full model sequence is therefore best read as a sensitivity bracket; the full-model coefficient is not a causal peer effect.

Policy evidence is mixed partly because policy definition, implementation, and enforcement differ. In English administrative data, Beland and Murphy reported a 0.07-SD post-ban increase in test performance, concentrated among previously low-achieving students [19]. Rau’s PISA 2022 analysis associated stricter restrictions with less classroom distraction and anxiety, with test-score gains when bans were effectively enforced [8]. By contrast, the 30-school SMART Schools study found that restrictive policies reduced in-school phone and social-media time but were not associated with better mental well-being or lower overall use [69]. Our non-significant peer-distraction-by-ban interaction is not a policy-effect estimate: the PISA variable records nominal premises-wide status, not enforcement or compliance. Likewise, our ESCS moderation does not replicate Beland and Murphy’s low-achievement subgroup because prior achievement and socioeconomic status are different modifiers and the designs are not comparable.

The secondary life-satisfaction result adds a level-specific distinction. Its null school-level association is compatible with SMART Schools [69], where restrictive policy was not associated with better mental well-being. Individual-level associations remained negative.

Large-scale reviews describe small or heterogeneous links between adolescents’ digital exposure and well-being [57–59]. However, the complete-case sample fell to 67,748 students. Selection into that analytic sample, differences between a single life-satisfaction item and multidimensional well-being, and the cross-sectional design preclude interpreting the null as evidence that the shared school environment is irrelevant to well-being.

Limitations include a cross-sectional observational design; a single-item perception-based aggregate; substantial complete-case restriction; nominal phone-policy measurement; incomplete creative-thinking coverage; and some screening diagnostics without full Fay’s BRR inference. Future studies need repeated or quasi-experimental designs, direct observation, validated multi-item measures, enforcement data, and prespecified causal diagrams. The most defensible implication is therefore not that peer distraction has been proven to cause learning loss, but that school digital-governance research should measure the shared attention environment and its implementation context directly.

## CONCLUSIONS

In a large cross-national PISA 2022 file, school-level leave-one-out peer digital distraction was associated with lower mathematics and creative-thinking performance in complete-case adjusted models. The association persisted across sensitivity analyses and was not mirrored by a significant school-level association with life satisfaction. These findings identify a robust observational association between peer digital-distraction climate and learning outcomes; they do not establish causality or evaluate the effectiveness of phone-ban policies.

## Additional material


Online Supplementary Document

## Data Availability

**Data availability:** PISA 2022 public-use data are available from the OECD PISA database. The present analysis used the PISA 2022 student questionnaire file, school questionnaire file, and creative-thinking data file. Country/economy grouping metadata for World Bank income group and UN M49 region are available from the World Bank and United Nations Statistics Division sources cited in the manuscript.
