# Supplementary material for: School-level peer digital distraction, mathematics performance, creative thinking, and adolescent well-being across 80 education systems: global evidence from PISA 2022
Source: J Glob Health. 2026 Nov 6;16:04351. doi: 10.7189/jogh.16.04351 (PMC13741168; doi:10.7189/jogh.16.04351)
Supplement: Supplementary file 1 [file jogh-16-04351-s001.pdf]

Supplement to: Sung CM, Lin CH, Pan HH, Lin KF, Ho YC. School-level peer digital distraction, mathematics performance, creative thinking, and adolescent well-being across 80 education systems: global evidence from PISA 2022. J Glob Health. 2026; 16:04351.

Supplementary material

Table S1. Adherence to JoGH GRABDROP Guidelines

GRABDROP item	Response
1. Papers by each co-author in the previous 3 years based on secondary analysis of a big data repository	Chien-Mei Sung: No peer-reviewed paper meeting GRABDROP item 1 criteria was identified.<br>Chia-Huei Lin: No peer-reviewed paper meeting GRABDROP item 1 criteria was identified.<br>Hsueh-Hsing Pan: No peer-reviewed paper meeting GRABDROP item 1 criteria was identified.<br>Ke-Feng Lin: Three qualifying peer-reviewed papers were identified:<br>1) Sun LC, Lee CC, Ke HY, Wei CY, Lin KF, Lin SS, Hsiu H, Chen PN. Deep learning for the classification of atrial fibrillation using wavelet transform-based visual images. BMC Med Inform Decis Mak. 2022;22(Suppl 5):349; published online 21 January 2025. doi:10.1186/s12911-025-02872-5.<br>2) Sun LC, Tseng CW, Lin KF, Chen PN. Hybrid preprocessing and ensemble classification for enhanced detection of Parkinson's disease using multiple speech signal databases. Digit Health. 2025;11:20552076251352941. doi:10.1177/20552076251352941.<br>3) Tseng CW, Sun LC, Lin KF, Chen PN. Enhancing coronary heart disease diagnosis: comparative analysis of data pre-processing techniques and machine learning models using clinical medical records. Health Informatics J. 2025;31(3):14604582251366160. doi:10.1177/14604582251366160.<br>These studies secondarily analysed, respectively, the public PhysioNet/Computing in Cardiology Challenge 2017 ECG repository; the public MIU, UEX, and UCI Parkinson disease speech datasets; and the public Heart Failure and Cleveland Heart Disease clinical-record datasets.<br>Yen-Chung Ho: No peer-reviewed paper meeting GRABDROP item 1 criteria was identified.
2. Key elements of study design and datasets that make the study original	The study uses public-use PISA 2022 student, school, and creative-thinking files; constructs a weighted leave-one-out school-level peer digital-distraction exposure; links it to mathematics plausible values, creative-thinking plausible values, and life satisfaction; applies PISA final and BRR-Fay replicate weights; and separates primary, sensitivity, heterogeneity, and screening analyses.
3. Publications addressing similar research questions in the same dataset	Closest same-dataset sources include OECD PISA 2022 reports on learning, disruption, digital resources, and creative thinking, and Rau's 2025 IDB working paper on school cellphone restrictions. The present study differs by focusing on a leave-one-out school-level peer-distraction climate rather than nominal restriction policies.
4. Multiple testing and statistical threshold	Confirmatory interpretation was limited to the prespecified school-level peer-distraction association for mathematics and creative thinking. Sensitivity, heterogeneity, contextual stratification, and demographic/SES-adjusted analyses were interpreted as robustness or exploratory analyses. P values are reported, but additional causal or policy-effect claims are not based on multiple secondary tests.
5. Use of AI chatbots	Chat GPT was used for English-language editing, wording refinement and journal-format polishing. Authors reviewed all AI-assisted edits and accept responsibility for the manuscript.

Table S2. Measurement, Missing-Data, and Collinearity Diagnostics

Domain	Diagnostic finding
Exposure support	Weighted mean = 1.951; weighted SD = 0.439; p5 = 1.235, median = 1.950, p95 = 2.688. One response-category contrast is approximately 2.28 weighted SDs.
Per-SD interpretation	Formal BRR/PV alternative-exposure analyses: mathematics B = -11.31 (95% CI -14.07 to -8.55); creative thinking B = -1.19 (95% CI -1.58 to -0.80), both p < .001.
School aggregation reliability	For schools with at least two valid reports: ICC(1) = 0.123; ICC(2) / reliability at unbalanced kbar = 0.726; school-level reliability IQR = 0.628-0.779.
Individual report vs school LOO mean	Weighted Pearson r = 0.303; weighted Spearman rho = 0.315 among students with both peer variables.
Complete-case samples	PV1 complete-case samples: mathematics n = 77,216 (weighted N = 3,071,053); creative thinking n = 65,222 (weighted N = 2,505,922); life satisfaction n = 67,748 (weighted N = 2,915,653).
Collinearity	Maximum VIF among main digital covariates = 2.51; maximum absolute pairwise correlation = 0.769; condition number = 9.224.

Domain	Diagnostic finding
Policy-enforcement proxy audit	Candidate PISA items captured attitudes, perceived teacher digital capacity, or general classroom climate, not actual phone-ban enforcement, compliance, or teacher monitoring practice; they were not promoted to main moderation analyses.
Source files	Detailed CSV outputs are retained in the revision diagnostics folder; key values are summarized here for manuscript transparency.

This supplementary file preserves the full cross-national classifications, sensitivity analyses, secondary analyses, and figures not retained in the JoGH main-text table/figure allowance. Table and figure numbering follows JoGH supplementary conventions.

Supplementary Figures

Figure S1. Conceptual model of peer digital distraction as a classroom externality.

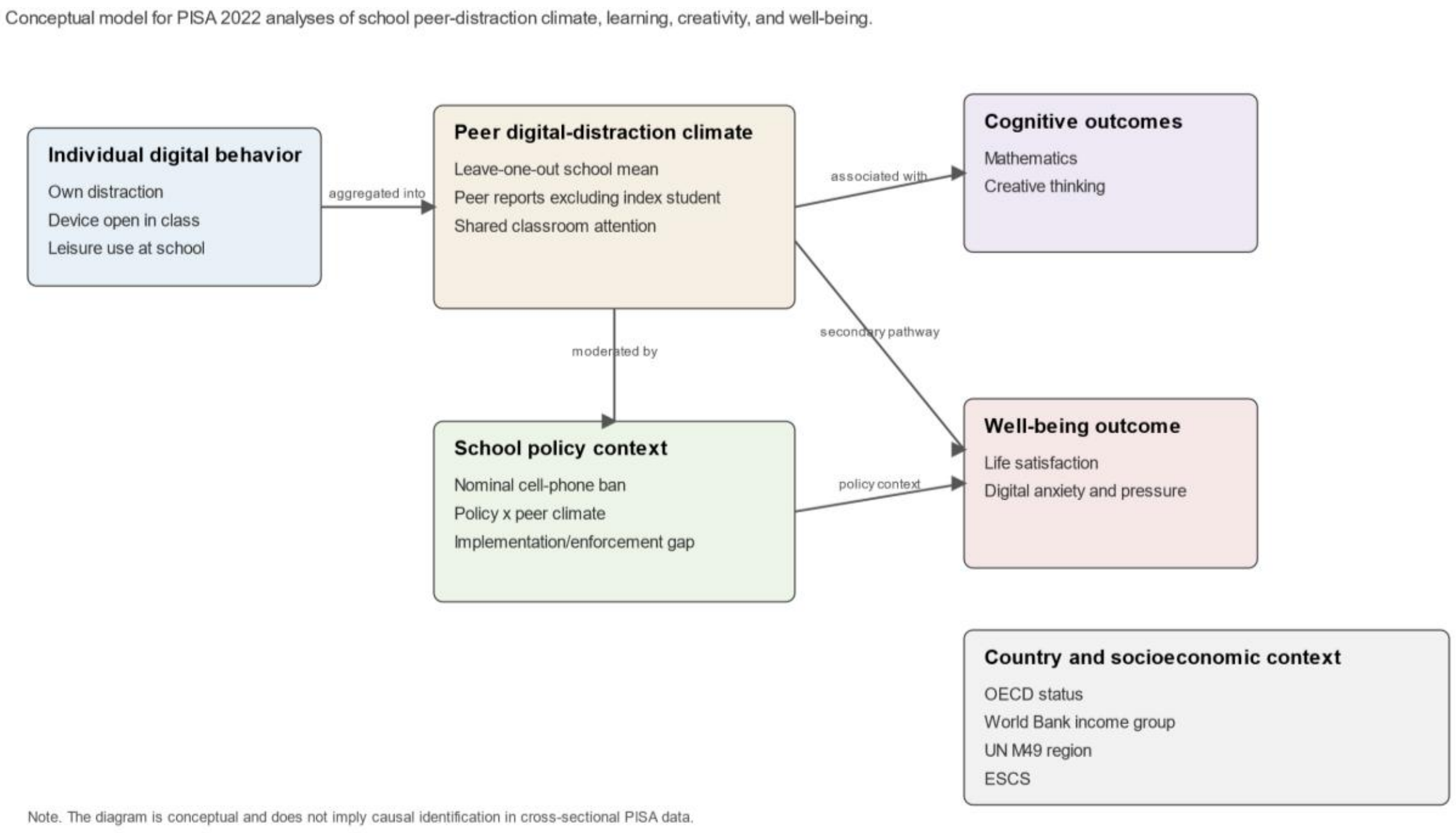

Figure S2. Core heterogeneity forest plot showing interaction estimates by OECD status and ESCS.

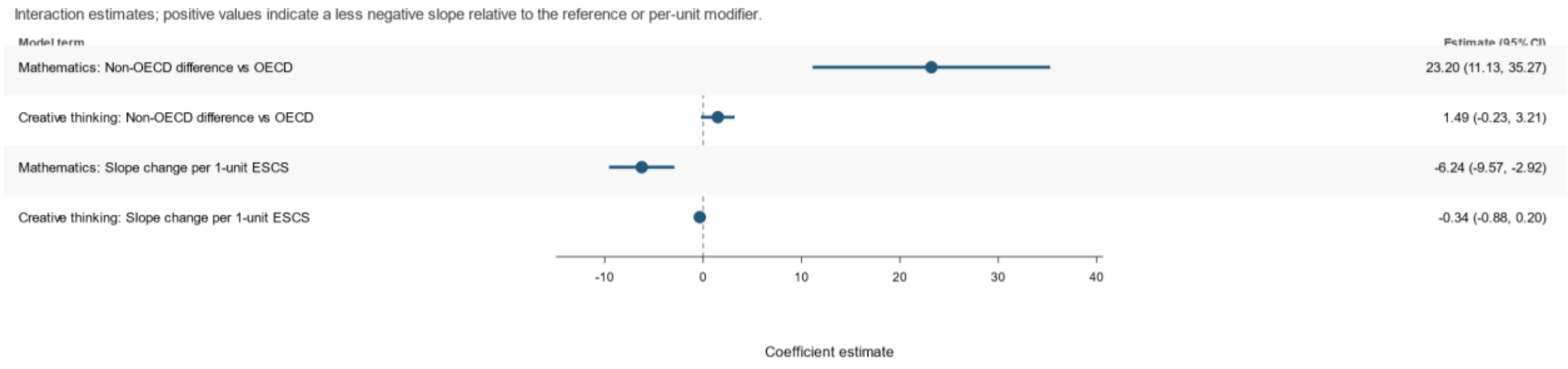

Figure S3. Country/economy phone-policy prevalence and peer-distraction climate.

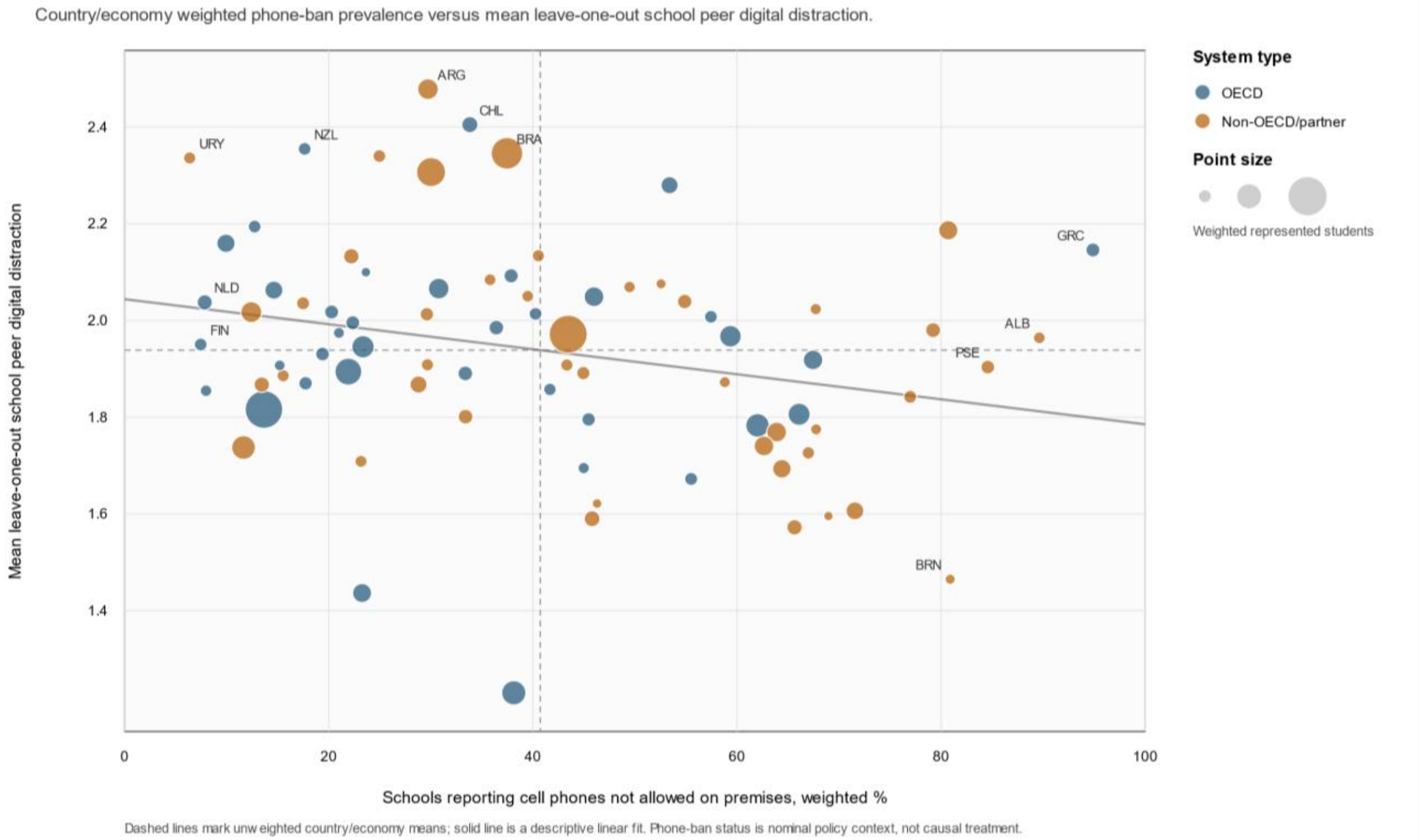

Supplementary Tables and Notes

Supplementary Material

All formal inferential sensitivity and heterogeneity tables used local IBM SPSS Statistics 24, PISA final student weights, and BRR-Fay replicate weights unless explicitly labelled as screening. Screening tables use final student weights and all 10 plausible values for point-estimate diagnostics or prioritization of future full BRR-Fay verification.

Table S3. Country/Economy Coverage and Classification

This table documents the cross-national scope underlying Table 1 and the country-group heterogeneity analyses.

Table S3 Panel A. Country/economy classification and creative-thinking coverage

PISA code	Country/economy	OECD status	World Bank income group	World Bank region	UN M49 region	UN M49 subregion	Creative-thinking PVs
ALB	Albania	Non-OECD/partner	Upper middle income	Europe & Central Asia	Europe	Southern Europe	Yes
ARE	United Arab Emirates	Non-OECD/partner	High income	Middle East, North Africa, Afghanistan & Pakistan	Asia	Western Asia	Yes
ARG	Argentina	Non-OECD/partner	Upper middle income	Latin America & Caribbean	Americas	South America	No
AUS	Australia	OECD	High income	East Asia & Pacific	Oceania	Australia and New Zealand	Yes
AUT	Austria	OECD	High income	Europe & Central Asia	Europe	Western Europe	No
BEL	Belgium	OECD	High income	Europe & Central Asia	Europe	Western Europe	Yes
BGR	Bulgaria	Non-OECD/partner	High income	Europe & Central Asia	Europe	Eastern Europe	Yes
BRA	Brazil	Non-OECD/partner	Upper middle income	Latin America & Caribbean	Americas	South America	Yes
BRN	Brunei Darussalam	Non-OECD/partner	High income	East Asia & Pacific	Asia	South-eastern Asia	Yes
CAN	Canada	OECD	High income	North America	Americas	Northern America	Yes
CHE	Switzerland	OECD	High income	Europe & Central Asia	Europe	Western Europe	No
CHL	Chile	OECD	High income	Latin America & Caribbean	Americas	South America	Yes
COL	Colombia	OECD	Upper middle income	Latin America & Caribbean	Americas	South America	Yes
CRI	Costa Rica	OECD	High income	Latin America & Caribbean	Americas	Central America	Yes
CZE	Czechia	OECD	High income	Europe & Central Asia	Europe	Eastern Europe	Yes
DEU	Germany	OECD	High income	Europe & Central Asia	Europe	Western Europe	Yes
DNK	Denmark	OECD	High income	Europe & Central Asia	Europe	Northern Europe	Yes
DOM	Dominican Republic	Non-OECD/partner	Upper middle income	Latin America & Caribbean	Americas	Caribbean	Yes

PISA code	Country/economy	OECD status	World Bank income group	World Bank region	UN M49 region	UN M49 subregion	Creative-thinking PVs
ESP	Spain	OECD	High income	Europe & Central Asia	Europe	Southern Europe	Yes
EST	Estonia	OECD	High income	Europe & Central Asia	Europe	Northern Europe	Yes
FIN	Finland	OECD	High income	Europe & Central Asia	Europe	Northern Europe	Yes
FRA	France	OECD	High income	Europe & Central Asia	Europe	Western Europe	Yes
GBR	United Kingdom	OECD	High income	Europe & Central Asia	Europe	Northern Europe	No
GEO	Georgia	Non-OECD/partner	Upper middle income	Europe & Central Asia	Asia	Western Asia	No
GRC	Greece	OECD	High income	Europe & Central Asia	Europe	Southern Europe	Yes
GTM	Guatemala	Non-OECD/partner	Upper middle income	Latin America & Caribbean	Americas	Central America	No
HKG	Hong Kong SAR, China	Non-OECD/partner	High income	East Asia & Pacific	Asia	Eastern Asia	Yes
HRV	Croatia	Non-OECD/partner	High income	Europe & Central Asia	Europe	Southern Europe	Yes
HUN	Hungary	OECD	High income	Europe & Central Asia	Europe	Eastern Europe	Yes
IDN	Indonesia	Non-OECD/partner	Upper middle income	East Asia & Pacific	Asia	South-eastern Asia	Yes
IRL	Ireland	OECD	High income	Europe & Central Asia	Europe	Northern Europe	No
ISL	Iceland	OECD	High income	Europe & Central Asia	Europe	Northern Europe	Yes
ISR	Israel	OECD	High income	Middle East, North Africa, Afghanistan & Pakistan	Asia	Western Asia	Yes
ITA	Italy	OECD	High income	Europe & Central Asia	Europe	Southern Europe	Yes
JAM	Jamaica	Non-OECD/partner	Upper middle income	Latin America & Caribbean	Americas	Caribbean	Yes
JOR	Jordan	Non-OECD/partner	Lower middle income	Middle East, North Africa, Afghanistan & Pakistan	Asia	Western Asia	Yes
JPN	Japan	OECD	High income	East Asia & Pacific	Asia	Eastern Asia	No
KAZ	Kazakhstan	Non-OECD/partner	Upper middle income	Europe & Central Asia	Asia	Central Asia	Yes
KHM	Cambodia	Non-OECD/partner	Lower middle income	East Asia & Pacific	Asia	South-eastern Asia	No
KOR	Korea, Rep.	OECD	High income	East Asia & Pacific	Asia	Eastern Asia	Yes
KSV	Kosovo	Non-OECD/partner	Upper middle income	Europe & Central Asia	Europe	Southern Europe	No

PISA code	Country/economy	OECD status	World Bank income group	World Bank region	UN M49 region	UN M49 subregion	Creative-thinking PVs
LTU	Lithuania	OECD	High income	Europe & Central Asia	Europe	Northern Europe	Yes
LVA	Latvia	OECD	High income	Europe & Central Asia	Europe	Northern Europe	Yes
MAC	Macao SAR, China	Non-OECD/partner	High income	East Asia & Pacific	Asia	Eastern Asia	Yes
MAR	Morocco	Non-OECD/partner	Lower middle income	Middle East, North Africa, Afghanistan & Pakistan	Africa	Northern Africa	Yes
MDA	Moldova	Non-OECD/partner	Upper middle income	Europe & Central Asia	Europe	Eastern Europe	Yes
MEX	Mexico	OECD	Upper middle income	Latin America & Caribbean	Americas	Central America	Yes
MKD	North Macedonia	Non-OECD/partner	Upper middle income	Europe & Central Asia	Europe	Southern Europe	Yes
MLT	Malta	Non-OECD/partner	High income	Middle East, North Africa, Afghanistan & Pakistan	Europe	Southern Europe	Yes
MNE	Montenegro	Non-OECD/partner	Upper middle income	Europe & Central Asia	Europe	Southern Europe	No
MNG	Mongolia	Non-OECD/partner	Upper middle income	East Asia & Pacific	Asia	Eastern Asia	Yes
MYS	Malaysia	Non-OECD/partner	Upper middle income	East Asia & Pacific	Asia	South-eastern Asia	Yes
NLD	Netherlands	OECD	High income	Europe & Central Asia	Europe	Western Europe	Yes
NOR	Norway	OECD	High income	Europe & Central Asia	Europe	Northern Europe	No
NZL	New Zealand	OECD	High income	East Asia & Pacific	Oceania	Australia and New Zealand	Yes
PAN	Panama	Non-OECD/partner	High income	Latin America & Caribbean	Americas	Central America	Yes
PER	Peru	Non-OECD/partner	Upper middle income	Latin America & Caribbean	Americas	South America	Yes
PHL	Philippines	Non-OECD/partner	Lower middle income	East Asia & Pacific	Asia	South-eastern Asia	Yes
POL	Poland	OECD	High income	Europe & Central Asia	Europe	Eastern Europe	Yes
PRT	Portugal	OECD	High income	Europe & Central Asia	Europe	Southern Europe	Yes
PRY	Paraguay	Non-OECD/partner	Upper middle income	Latin America & Caribbean	Americas	South America	No
PSE	West Bank and Gaza	Non-OECD/partner	Lower middle income	Middle East, North Africa, Afghanistan & Pakistan	Asia	Western Asia	Yes

PISA code	Country/economy	OECD status	World Bank income group	World Bank region	UN M49 region	UN M49 subregion	Creative-thinking PVs
QAT	Qatar	Non-OECD/partner	High income	Middle East, North Africa, Afghanistan & Pakistan	Asia	Western Asia	Yes
QAZ	Baku (Azerbaijan)	Non-OECD/partner	Upper middle income	Europe & Central Asia	Asia	Western Asia	Yes
QUR	Ukrainian regions (18 of 27)	Non-OECD/partner	Upper middle income	Europe & Central Asia	Europe	Eastern Europe	Yes
ROU	Romania	Non-OECD/partner	High income	Europe & Central Asia	Europe	Eastern Europe	Yes
SAU	Saudi Arabia	Non-OECD/partner	High income	Middle East, North Africa, Afghanistan & Pakistan	Asia	Western Asia	Yes
SGP	Singapore	Non-OECD/partner	High income	East Asia & Pacific	Asia	South-eastern Asia	Yes
SLV	El Salvador	Non-OECD/partner	Upper middle income	Latin America & Caribbean	Americas	Central America	Yes
SRB	Serbia	Non-OECD/partner	Upper middle income	Europe & Central Asia	Europe	Southern Europe	Yes
SVK	Slovak Republic	OECD	High income	Europe & Central Asia	Europe	Eastern Europe	Yes
SVN	Slovenia	OECD	High income	Europe & Central Asia	Europe	Southern Europe	Yes
SWE	Sweden	OECD	High income	Europe & Central Asia	Europe	Northern Europe	No
TAP	Chinese Taipei	Non-OECD/partner	High income	East Asia & Pacific	Asia	Eastern Asia	Yes
THA	Thailand	Non-OECD/partner	Upper middle income	East Asia & Pacific	Asia	South-eastern Asia	Yes
TUR	Turkiye	OECD	Upper middle income	Europe & Central Asia	Asia	Western Asia	No
URY	Uruguay	Non-OECD/partner	High income	Latin America & Caribbean	Americas	South America	Yes
USA	United States	OECD	High income	North America	Americas	Northern America	No
UZB	Uzbekistan	Non-OECD/partner	Lower middle income	Europe & Central Asia	Asia	Central Asia	Yes
VNM	Viet Nam	Non-OECD/partner	Lower middle income	East Asia & Pacific	Asia	South-eastern Asia	No

Table S3 Panel B. Country/economy weighted population, exposure and policy summaries

PISA code	Country/economy	Weighted represented students	Mean peer distraction	Mean school peer LOO	Phone-ban schools, %	Peer-burden quartile	Phone-ban prevalence quartile
ALB	Albania	28,426	1.93	1.96	89.6	Q3	Q4

PISA code	Country/economy	Weighted represented students	Mean peer distraction	Mean school peer LOO	Phone-ban schools, %	Peer-burden quartile	Phone-ban prevalence quartile
ARE	United Arab Emirates	60,765	1.81	1.84	77.0	Q2	Q4
ARG	Argentina	596,301	2.47	2.48	29.7	Q4	Q2
AUS	Australia	265,196	2.28	2.28	53.4	Q4	Q3
AUT	Austria	76,153	1.83	1.87	17.7	Q2	Q1
BEL	Belgium	128,642	1.97	1.99	36.4	Q3	Q2
BGR	Bulgaria	53,421	2.30	2.34	25.0	Q4	Q2
BRA	Brazil	2,262,972	2.34	2.35	37.5	Q4	Q2
BRN	Brunei Darussalam	5,980	1.46	1.46	80.9	Q1	Q4
CAN	Canada	357,911	2.15	2.16	9.9	Q4	Q1
CHE	Switzerland	75,696	1.79	1.80	45.5	Q1	Q3
CHL	Chile	214,108	2.39	2.40	33.8	Q4	Q2
COL	Colombia	586,683	2.05	2.07	30.8	Q4	Q2
CRI	Costa Rica	57,250	2.19	2.19	12.7	Q4	Q1
CZE	Czechia	100,266	2.01	2.02	20.3	Q3	Q1
DEU	Germany	681,399	1.96	1.97	59.4	Q3	Q4
DNK	Denmark	56,909	2.01	2.01	40.3	Q3	Q3
DOM	Dominican Republic	121,876	1.98	2.04	54.9	Q3	Q3
ESP	Spain	459,029	1.91	1.92	67.4	Q2	Q4
EST	Estonia	13,345	1.90	1.91	15.2	Q2	Q1
FIN	Finland	58,955	1.94	1.95	7.5	Q3	Q1
FRA	France	781,286	1.93	1.95	23.4	Q2	Q2
GBR	United Kingdom	731,225	1.80	1.81	66.1	Q1	Q4
GEO	Georgia	40,416	1.89	1.91	29.7	Q2	Q2
GRC	Greece	98,087	2.13	2.15	94.9	Q4	Q4
GTM	Guatemala	168,484	1.55	1.57	65.6	Q1	Q4
HKG	Hong Kong SAR, China	48,246	1.72	1.73	67.0	Q1	Q4
HRV	Croatia	35,033	1.69	1.71	23.2	Q1	Q2
HUN	Hungary	87,990	1.92	1.93	19.4	Q2	Q1
IDN	Indonesia	3,790,846	1.97	1.97	43.5	Q3	Q3
IRL	Ireland	65,497	1.67	1.67	55.5	Q1	Q3

PISA code	Country/economy	Weighted represented students	Mean peer distraction	Mean school peer LOO	Phone-ban schools, %	Peer-burden quartile	Phone-ban prevalence quartile
ISL	Iceland	4,351	2.09	2.10	23.6	Q4	Q2
ISR	Israel	132,475	1.88	1.89	33.4	Q2	Q2
ITA	Italy	496,263	2.04	2.05	46.0	Q3	Q3
JAM	Jamaica	25,495	2.01	2.07	49.5	Q3	Q3
JOR	Jordan	144,269	1.93	1.98	79.2	Q3	Q4
JPN	Japan	1,021,370	1.23	1.23	38.1	Q1	Q3
KAZ	Kazakhstan	272,446	1.87	1.87	28.8	Q2	Q2
KHM	Cambodia	126,409	1.80	1.80	33.4	Q2	Q2
KOR	Korea, Rep.	428,012	1.44	1.44	23.3	Q1	Q2
KSV	Kosovo	21,045	2.00	2.02	67.7	Q3	Q4
LTU	Lithuania	24,251	1.84	1.85	8.0	Q2	Q1
LVA	Latvia	16,833	1.97	1.97	21.0	Q3	Q1
MAC	Macao SAR, China	4,423	1.62	1.62	46.3	Q1	Q3
MAR	Morocco	454,986	2.17	2.19	80.7	Q4	Q4
MDA	Moldova	28,879	2.08	2.08	35.8	Q4	Q2
MEX	Mexico	1,393,727	1.89	1.89	21.9	Q2	Q1
MKD	North Macedonia	16,548	1.85	1.87	58.8	Q2	Q3
MLT	Malta	3,955	1.58	1.60	69.0	Q1	Q4
MNE	Montenegro	6,340	2.07	2.08	52.6	Q4	Q3
MNG	Mongolia	40,828	2.14	2.13	40.5	Q4	Q3
MYS	Malaysia	390,447	1.69	1.69	64.4	Q1	Q4
NLD	Netherlands	155,987	2.03	2.04	7.8	Q3	Q1
NOR	Norway	58,970	2.00	2.01	57.4	Q3	Q3
NZL	New Zealand	56,382	2.35	2.35	17.6	Q4	Q1
PAN	Panama	42,090	1.88	1.91	43.3	Q2	Q3
PER	Peru	499,075	1.76	1.77	63.9	Q1	Q4
PHL	Philippines	1,782,896	2.31	2.31	30.0	Q4	Q2
POL	Poland	341,562	2.05	2.06	14.6	Q4	Q1
PRT	Portugal	96,607	2.00	2.00	22.4	Q3	Q2
PRY	Paraguay	81,004	2.00	2.01	29.6	Q3	Q2

PISA code	Country/economy	Weighted represented students	Mean peer distraction	Mean school peer LOO	Phone-ban schools, %	Peer-burden quartile	Phone-ban prevalence quartile
PSE	West Bank and Gaza	88,383	1.87	1.90	84.6	Q2	Q4
QAT	Qatar	18,348	1.72	1.78	67.8	Q1	Q4
QAZ	Baku (Azerbaijan)	30,529	2.02	2.05	39.5	Q3	Q3
QUR	Ukrainian regions (18 of 27)	165,592	1.86	1.87	13.4	Q2	Q1
ROU	Romania	162,019	2.12	2.13	22.2	Q4	Q2
SAU	Saudi Arabia	317,452	1.57	1.61	71.6	Q1	Q4
SGP	Singapore	41,958	1.88	1.89	15.5	Q2	Q1
SLV	El Salvador	68,170	1.86	1.89	45.0	Q2	Q3
SRB	Serbia	59,250	2.03	2.04	17.5	Q3	Q1
SVK	Slovak Republic	47,453	1.84	1.86	41.7	Q2	Q3
SVN	Slovenia	18,850	1.69	1.69	45.0	Q1	Q3
SWE	Sweden	108,499	2.08	2.09	37.9	Q4	Q2
TAP	Chinese Taipei	190,787	1.59	1.59	45.8	Q1	Q3
THA	Thailand	604,573	2.01	2.02	12.4	Q3	Q1
TUR	Turkiye	933,402	1.78	1.78	62.0	Q1	Q4
URY	Uruguay	40,778	2.32	2.34	6.4	Q4	Q1
USA	United States	3,661,328	1.82	1.82	13.6	Q2	Q1
UZB	Uzbekistan	482,059	1.75	1.74	62.6	Q1	Q4
VNM	Viet Nam	939,459	1.74	1.74	11.6	Q1	Q1

Notes:

Weighted represented students are based on PISA final student weights in the corrected analytic file.

Mean peer distraction is the weighted country/economy mean of the recoded individual peer-distraction item; higher values indicate more frequent peer distraction.

Mean school peer LOO is the weighted country/economy mean of the leave-one-out school peer-distraction exposure.

Phone-ban schools, % is the weighted proportion of students in schools reporting that cell phones are not allowed on school premises (SC190Q02JA).

Across the 80 country/economy profiles, the unweighted mean of these student-weighted phone-ban prevalence estimates was 40.7%.

World Bank income group and region use the project metadata file documented in secondary_sensitivity_analyses/metadata/classification_sources.md; UN regions use UN M49 broad region and subregion.

Peer-burden and phone-ban prevalence quartiles were used for contextual stratification screening.

Table S4. School Peer-Count Threshold Sensitivity

Outcome coefficients are for leave-one-out school peer digital distraction. All models use corrected phone-policy variable SC190Q02JA, country/economy fixed effects, 10 plausible values, 80 BRR-Fay replicate weights, and the same adjusted covariate set as the primary model.

Outcome	School peer-count sample	Estimate	SE	95% CI	P	PVs	Replicates
Creative thinking	Full sample	-3.81	0.53	-4.85 to -2.76	<0.001	10	80
Creative thinking	school_n_peer >= 5	-4.52	0.55	-5.60 to -3.44	<0.001	10	80
Creative thinking	school_n_peer >= 10	-4.69	0.58	-5.83 to -3.55	<0.001	10	80
Creative thinking	school_n_peer >= 15	-5.06	0.59	-6.22 to -3.90	<0.001	10	80
Mathematics	Full sample	-34.28	3.95	-42.02 to -26.54	<0.001	10	80
Mathematics	school_n_peer >= 5	-39.81	3.57	-46.82 to -32.81	<0.001	10	80
Mathematics	school_n_peer >= 10	-41.41	3.75	-48.76 to -34.06	<0.001	10	80
Mathematics	school_n_peer >= 15	-44.69	3.76	-52.06 to -37.32	<0.001	10	80

Table S5. Model-Adjustment Sensitivity

Outcome-specific estimates for the school-level leave-one-out peer digital-distraction exposure. All models used PISA final student weights, 10 plausible values, and 80 BRR-Fay replicate weights in local IBM SPSS Statistics 24.

Outcome	Model	B	SE	95% CI	p	PVs	Replicates per PV
Mathematics	Minimal country/economy fixed-effects model	-38.53	1.90	[-42.25, -34.80]	<.001	10	80
Mathematics	Student digital covariate model	-31.13	2.02	[-35.09, -27.17]	<.001	10	80
Mathematics	Full adjusted model with psychosocial digital covariates and phone-policy interaction	-34.28	3.95	[-42.02, -26.54]	<.001	10	80
Creative thinking	Minimal country/economy fixed-effects model	-4.57	0.29	[-5.14, -4.00]	<.001	10	80
Creative thinking	Student digital covariate model	-3.73	0.40	[-4.51, -2.95]	<.001	10	80
Creative thinking	Full adjusted model with psychosocial digital covariates and phone-policy interaction	-3.81	0.53	[-4.85, -2.76]	<.001	10	80

Note. The student digital covariate model adjusts for individual perceived peer distraction, own digital distraction in mathematics lessons, device open in class, and in-school leisure digital-resource use. The full adjusted model additionally includes digital anxiety, pressure to be online/respond in class, school cell-phone ban status, and the school peer-distraction by phone-ban interaction.

Table S6. Sample-Restriction Sensitivity

Outcome-specific estimates for the school-level leave-one-out peer digital-distraction exposure. All models used the full adjusted covariate specification, PISA final student weights, 10 plausible values, and 80 BRR-Fay replicate weights in local IBM SPSS Statistics 24.

Outcome	Sample restriction	B	SE	95% CI	p	PVs	Replicates per PV
Mathematics	Full analytic sample	-34.28	3.95	[-42.02, -26.54]	<.001	10	80
Mathematics	OECD systems only	-44.07	4.02	[-51.95, -36.19]	<.001	10	80

Outcome	Sample restriction	B	SE	95% CI	p	PVs	Replicates per PV
Mathematics	Non-OECD/partner systems only	-23.18	6.20	[-35.34, -11.02]	<.001	10	80
Mathematics	Creative-thinking participating systems only	-31.69	4.17	[-39.85, -23.52]	<.001	10	80
Creative thinking	Full analytic sample	-3.81	0.53	[-4.85, -2.76]	<.001	10	80
Creative thinking	OECD systems only	-4.72	0.50	[-5.69, -3.74]	<.001	10	80
Creative thinking	Non-OECD/partner systems only	-3.00	0.90	[-4.76, -1.23]	<.001	10	80
Creative thinking	Creative-thinking participating systems only	-3.81	0.53	[-4.85, -2.76]	<.001	10	80

Note. Creative-thinking outcomes are available only in creative-thinking participating systems; therefore, the full creative-thinking model and the creative-participating-system restriction are expected to be identical or nearly identical. OECD coding follows the PISA OECD indicator.

Table S7. Phone-Policy Complete-Case Sensitivity

Outcome-specific estimates for the school-level leave-one-out peer digital-distraction exposure. The comparison tests whether restricting models to students with nonmissing school phone-policy status materially changes the school peer-distraction slope before phone-policy terms are added to the full model.

Outcome	Model	Sample	B	SE	95% CI	p	PVs	Replicates per PV	Complete
Mathematics	Minimal country/economy fixed-effects model	Unrestricted	-38.53	1.90	[-42.25, -34.80]	<.001	10	80	Yes
Mathematics	Minimal country/economy fixed-effects model	Phone-policy complete	-38.43	1.85	[-42.07, -34.79]	<.001	10	80	Yes
Mathematics	Student digital covariate model	Unrestricted	-31.13	2.02	[-35.09, -27.17]	<.001	10	80	Yes
Mathematics	Student digital covariate model	Phone-policy complete	-31.50	2.12	[-35.65, -27.35]	<.001	10	80	Yes
Mathematics	Full adjusted model with phone-policy terms	Phone-policy complete by model design	-34.28	3.95	[-42.02, -26.54]	<.001	10	80	Yes
Creative thinking	Minimal country/economy fixed-effects model	Unrestricted	-4.57	0.29	[-5.14, -4.00]	<.001	10	80	Yes
Creative thinking	Minimal country/economy fixed-effects model	Phone-policy complete	-4.73	0.29	[-5.30, -4.16]	<.001	10	80	Yes
Creative thinking	Student digital covariate model	Unrestricted	-3.73	0.40	[-4.51, -2.95]	<.001	10	80	Yes
Creative thinking	Student digital covariate model	Phone-policy complete	-3.80	0.40	[-4.60, -3.01]	<.001	10	80	Yes
Creative thinking	Full adjusted model with phone-policy terms	Phone-policy complete by model design	-3.81	0.53	[-4.85, -2.76]	<.001	10	80	Yes

Notes:

Phone-policy complete models used NOT MISSING(phone_ban_binary), where phone_ban_binary was derived from school questionnaire item SC190Q02JA.

The student digital covariate model adjusts for individual perceived peer distraction, own digital distraction in mathematics lessons, device open in class, and in-school leisure digital-resource use.

The full adjusted model additionally includes digital anxiety, pressure to be online/respond in class, school cell-phone ban status, and the school peer-distraction by phone-ban interaction.

Standard errors use PISA final and replicate weights with Fay's BRR and plausible-value pooling.

Table S8. Extreme Peer-Distraction-Burden Exclusion Sensitivity

Country/economy peer-distraction burden was defined as the country/economy weighted mean of the school-level leave-one-out peer digital-distraction exposure. Extreme systems were the lowest and highest 5% of the 80 systems, corresponding to four systems in each tail.

Outcome	Analysis	Excluded systems	Exposure B	SE	95% CI	p	Interaction with phone ban B	SE	95% CI	p	Complete
Mathematics	Primary full sample	None	-34.28	3.95	[-42.02, -26.54]	<.001	4.88	4.58	[-4.10, 13.85]	.287	Yes
Mathematics	Exclude lowest and highest 5% burden systems	JPN, KOR, BRN, GTM, BRA, NZL, CHL, ARG	-37.74	4.49	[-46.53, -28.94]	<.001	3.77	5.36	[-6.75, 14.28]	.483	Yes
Mathematics	Exclude highest 5% burden systems	BRA, NZL, CHL, ARG	-38.50	4.31	[-46.94, -30.06]	<.001	4.07	5.13	[-5.98, 14.12]	.427	Yes
Mathematics	Exclude lowest 5% burden systems	JPN, KOR, BRN, GTM	-33.46	3.91	[-41.12, -25.79]	<.001	4.46	4.55	[-4.45, 13.38]	.327	Yes
Creative thinking	Primary full sample	None	-3.81	0.53	[-4.85, -2.76]	<.001	0.55	0.72	[-0.87, 1.96]	.448	Yes
Creative thinking	Exclude lowest and highest 5% burden systems	JPN, KOR, BRN, GTM, BRA, NZL, CHL, ARG	-4.45	0.62	[-5.67, -3.24]	<.001	0.33	0.86	[-1.36, 2.03]	.700	Yes
Creative thinking	Exclude highest 5% burden systems	BRA, NZL, CHL, ARG	-4.51	0.60	[-5.69, -3.34]	<.001	0.36	0.82	[-1.25, 1.96]	.665	Yes
Creative thinking	Exclude lowest 5% burden systems	JPN, KOR, BRN, GTM	-3.74	0.54	[-4.81, -2.68]	<.001	0.52	0.76	[-0.98, 2.01]	.498	Yes

Notes:

Lowest 5% burden systems: JPN, KOR, BRN, GTM.

Highest 5% burden systems: BRA, NZL, CHL, ARG.

All exclusion models used the same full adjusted covariate specification as the primary model, including country/economy fixed effects, corrected phone-policy variable SC190Q02JA, and the school peer-distraction by phone-ban interaction.

Standard errors use PISA final and replicate weights with Fay's BRR and plausible-value pooling.

Table S9. Leave-One-Country/Economy-Out Influence Screening

This screening used local IBM SPSS Statistics 24 to estimate the full adjusted model after excluding one PISA country/economy at a time. Estimates use the final student weight and 10 plausible values; they are point-estimate influence diagnostics rather than BRR-based inference. Countries/economies are ranked by the absolute shift in the school-level leave-one-out peer-distraction coefficient relative to the full-sample final-weight plausible-value mean.

Mathematics

Full-sample final-weight PV mean: -34.28.

Leave-one-country/economy-out range: -37.78 to -31.01.

Largest absolute shift: 3.50 points.

Rank	Excluded system	LOO estimate	Shift from primary	Relative shift	Country peer burden	Weighted students
1	BRA	-37.78	-3.50	10.2%	2.35	2262972
2	TUR	-31.01	3.27	9.5%	1.78	933402
3	PHL	-32.65	1.62	4.7%	2.31	1782896
4	IDN	-35.41	-1.13	3.3%	1.97	3790846
5	MEX	-35.38	-1.10	3.2%	1.89	1393727
6	DEU	-33.33	0.95	2.8%	1.97	681399
7	FRA	-33.36	0.92	2.7%	1.95	781286
8	KOR	-33.53	0.75	2.2%	1.44	428012
9	NLD	-33.65	0.63	1.8%	2.04	155987
10	SAU	-34.85	-0.57	1.7%	1.61	317452

Creative thinking

Full-sample final-weight PV mean: -3.81.

Leave-one-country/economy-out range: -4.52 to -3.34.

Largest absolute shift: 0.71 points.

Rank	Excluded system	LOO estimate	Shift from primary	Relative shift	Country peer burden	Weighted students
1	BRA	-4.52	-0.71	18.6%	2.35	2262972
2	PHL	-3.34	0.47	12.2%	2.31	1782896
3	CAN	-3.92	-0.12	3.0%	2.16	357911
4	DEU	-3.70	0.11	2.8%	1.97	681399
5	FRA	-3.72	0.09	2.4%	1.95	781286
6	POL	-3.73	0.08	2.2%	2.06	341562
7	NLD	-3.74	0.07	1.8%	2.04	155987
8	ITA	-3.74	0.06	1.7%	2.05	496263
9	KOR	-3.75	0.06	1.6%	1.44	428012
10	ESP	-3.87	-0.06	1.6%	1.92	459029

Notes:

The table is intended to identify whether any single country/economy drives the primary point estimate.

Because this is a final-weight point-estimate screen, formal uncertainty remains based on the primary BRR/PV analyses and targeted BRR checks if needed.

The screened coefficient is the school-level leave-one-out peer digital-distraction exposure.

Table S10. Alternative Exposure-Definition Sensitivity

Outcome	Exposure definition	Scale	Exposure B	SE	95% CI	P	Interaction with school phone ban B	SE	95% CI	P	Complete
Mathematics	Leave-one-out school peer distraction	One response-category point on school leave-one-out mean	-34.28	3.95	-42.02 to -26.54	<.001	4.88	4.58	-4.10 to 13.85	.287	Yes
Mathematics	Leave-in school peer distraction	One response-category point on school mean including the index student	-36.90	3.81	-44.37 to -29.44	<.001	6.35	4.56	-2.59 to 15.29	.164	Yes
Mathematics	Within-country standardized leave-one-out peer distraction	One within-country SD	-11.31	1.41	-14.07 to -8.55	<.001	-0.72	1.85	-4.34 to 2.90	.697	Yes
Mathematics	Quartile score of leave-one-out peer distraction	One quartile-score increase, 1=lowest to 4=highest	-12.16	1.14	-14.40 to -9.93	<.001	2.04	1.53	-0.96 to 5.04	.183	Yes
Creative thinking	Leave-one-out school peer distraction	One response-category point on school leave-one-out mean	-3.81	0.53	-4.85 to -2.76	<.001	0.55	0.72	-0.87 to 1.96	.448	Yes
Creative thinking	Leave-in school peer distraction	One response-category point on school mean including the index student	-4.21	0.53	-5.25 to -3.17	<.001	1.11	0.73	-0.32 to 2.54	.127	Yes
Creative thinking	Within-country standardized leave-one-out peer distraction	One within-country SD	-1.19	0.20	-1.58 to -0.80	<.001	-0.46	0.33	-1.10 to 0.18	.160	Yes
Creative thinking	Quartile score of leave-one-out peer distraction	One quartile-score increase, 1=lowest to 4=highest	-1.39	0.17	-1.71 to -1.06	<.001	0.20	0.26	-0.31 to 0.71	.440	Yes

Notes:

Primary leave-one-out estimates are shown as the reference specification.

All alternative exposure models use the same covariate-adjusted specification as the primary models: country/economy fixed effects; individual perceived peer distraction; own digital distraction in mathematics lessons; device open in class; in-school leisure digital-resource use; digital anxiety; pressure to be online/respond in class; school cell-phone ban; and the exposure-by-phone-ban interaction.

Standard errors use PISA final and replicate weights with Fay's BRR and plausible-value pooling.

Interaction estimates test whether the exposure association differs between schools reporting a premises-wide cell-phone ban and schools without such a ban.

Table S11. Core Heterogeneity

All estimates use corrected phone-policy variable SC190Q02JA, country/economy fixed effects, 10 plausible values, 80 BRR-Fay replicate weights, and the same adjusted covariates as the primary model unless noted.

Analysis	Outcome	Term	Estimate	SE	95% CI	P	PVs	Replicates
ESCS heterogeneity	Creative thinking	ESCS main effect	3.89	0.55	2.82 to 4.97	<0.001	10	80
ESCS heterogeneity	Creative thinking	School peer distraction slope at ESCS = 0	-3.32	0.52	-4.34 to -2.31	<0.001	10	80
ESCS heterogeneity	Creative thinking	School peer distraction x ESCS	-0.34	0.28	-0.88 to 0.20	0.216	10	80
ESCS heterogeneity	Mathematics	ESCS main effect	37.58	3.45	30.82 to 44.33	<0.001	10	80
ESCS heterogeneity	Mathematics	School peer distraction slope at ESCS = 0	-32.66	3.79	-40.10 to -25.23	<0.001	10	80
ESCS heterogeneity	Mathematics	School peer distraction x ESCS	-6.24	1.69	-9.57 to -2.92	<0.001	10	80
OECD heterogeneity	Creative thinking	Non-OECD difference in school peer-distraction slope	1.49	0.88	-0.23 to 3.21	0.089	10	80
OECD heterogeneity	Creative thinking	School peer distraction slope in OECD systems	-4.65	0.49	-5.61 to -3.68	<0.001	10	80
OECD heterogeneity	Mathematics	Non-OECD difference in school peer-distraction slope	23.20	6.16	11.13 to 35.27	<0.001	10	80
OECD heterogeneity	Mathematics	School peer distraction slope in OECD systems	-46.21	3.69	-53.46 to -38.97	<0.001	10	80

Interpretation notes:

In the OECD model, the school peer-distraction coefficient is the slope for OECD systems. The non-OECD interaction is the difference in slope for non-OECD systems relative to OECD systems.

The implied non-OECD mathematics slope is approximately -23.01 (-46.21 + 23.20). The implied non-OECD creative-thinking slope is approximately -3.16 (-4.65 + 1.49). Formal CIs for these implied slopes require linear-combination covariance estimates and are not shown here.

In the ESCS model, the peer-distraction coefficient is the slope at ESCS = 0, and the interaction term indicates how the peer-distraction slope changes per 1-unit increase in ESCS.

Table S12. World Bank Income Group and UN M49 Region Heterogeneity

All models used corrected phone-policy variable SC190Q02JA, country/economy fixed effects, 10 plausible values, 80 BRR-Fay replicate weights, and the same adjusted covariates as the primary model.

Analysis	Outcome	Term	Estimate	SE	95% CI	P	PVs	Replicates
UN M49 broad region	Creative thinking	Africa difference vs Oceania	-1.00	2.27	-5.45 to 3.45	0.66	10	80
UN M49 broad region	Creative thinking	Americas difference vs Oceania	1.01	1.14	-1.21 to 3.24	0.372	10	80
UN M49 broad region	Creative thinking	Asia difference vs Oceania	-1.91	1.29	-4.42 to 0.61	0.138	10	80
UN M49 broad region	Creative thinking	Europe difference vs Oceania	-2.79	0.83	-4.42 to -1.16	<0.001	10	80
UN M49 broad region	Creative thinking	Reference-group school peer-distraction slope	-2.75	0.82	-4.37 to -1.13	<0.001	10	80
UN M49 broad region	Mathematics	Africa difference vs Oceania	2.77	15.02	-26.67 to 32.20	0.854	10	80
UN M49 broad region	Mathematics	Americas difference vs Oceania	13.30	8.61	-3.57 to 30.18	0.122	10	80
UN M49 broad region	Mathematics	Asia difference vs Oceania	-13.24	9.81	-32.46 to 5.98	0.177	10	80

Analysis	Outcome	Term	Estimate	SE	95% CI	P	PVs	Replicates
UN M49 broad region	Mathematics	Europe difference vs Oceania	-19.79	7.85	-35.18 to -4.40	0.012	10	80
UN M49 broad region	Mathematics	Reference-group school peer-distraction slope	-28.39	8.12	-44.30 to -12.48	<0.001	10	80
World Bank income group	Creative thinking	High income difference vs lower-middle income	2.30	1.48	-0.61 to 5.21	0.121	10	80
World Bank income group	Creative thinking	Upper-middle income difference vs lower-middle income	4.47	1.49	1.55 to 7.40	0.003	10	80
World Bank income group	Creative thinking	Reference-group school peer-distraction slope	-7.16	1.52	-10.15 to -4.17	<0.001	10	80
World Bank income group	Mathematics	High income difference vs lower-middle income	-6.12	8.28	-22.34 to 10.10	0.46	10	80
World Bank income group	Mathematics	Upper-middle income difference vs lower-middle income	5.84	8.13	-10.09 to 21.77	0.473	10	80
World Bank income group	Mathematics	Reference-group school peer-distraction slope	-35.52	8.40	-51.97 to -19.06	<0.001	10	80

Interpretation notes:

In the World Bank income model, lower-middle-income systems are the reference group. High-income and upper-middle-income rows are differences in the peer-distraction slope relative to lower-middle-income systems.

In the UN M49 broad-region model, Oceania is the reference group because of SPSS parameterization. Because Oceania includes only Australia and New Zealand in this PISA analytic file, broad-region contrasts should be interpreted as exploratory and are best placed in the supplement.

Formal confidence intervals for implied group-specific slopes require linear-combination covariance estimates and are not shown in this table.

Table S13. Contextual Stratification Screening

This table reports final-student-weight, 10-plausible-value screening point estimates from local IBM SPSS Statistics 24. These estimates are intended to prioritize future full BRR-Fay verification; they should not be interpreted as final design-based inferential tests.

Outcome	Context	Contrast	PV-mean B	PV range	PVs
Mathematics	Country peer-distraction burden quartile	Reference slope: Q4 highest country peer-distraction burden	-23.64	[-25.21, -20.82]	10
Mathematics	Country peer-distraction burden quartile	Q1 difference versus Q4 reference	-22.92	[-25.43, -20.96]	10
Mathematics	Country peer-distraction burden quartile	Q2 difference versus Q4 reference	-10.05	[-12.75, -7.90]	10
Mathematics	Country peer-distraction burden quartile	Q3 difference versus Q4 reference	-16.28	[-18.03, -12.92]	10
Mathematics	Country school phone-ban prevalence quartile	Reference slope: Q4 highest country phone-ban prevalence	-46.56	[-47.50, -44.62]	10
Mathematics	Country school phone-ban prevalence quartile	Q1 difference versus Q4 reference	13.31	[11.81, 14.94]	10
Mathematics	Country school phone-ban prevalence quartile	Q2 difference versus Q4 reference	17.57	[15.93, 19.42]	10
Mathematics	Country school phone-ban prevalence quartile	Q3 difference versus Q4 reference	13.62	[10.50, 18.96]	10
Mathematics	Student ESCS quartile within country/economy	Reference slope: Q4 highest within-country student ESCS	-32.27	[-33.80, -29.53]	10

Outcome	Context	Contrast	PV-mean B	PV range	PVs
Mathematics	Student ESCS quartile within country/economy	Q1 difference versus Q4 reference	11.33	[9.22, 13.70]	10
Mathematics	Student ESCS quartile within country/economy	Q2 difference versus Q4 reference	4.55	[2.70, 6.91]	10
Mathematics	Student ESCS quartile within country/economy	Q3 difference versus Q4 reference	-2.71	[-4.41, 0.24]	10
Mathematics	School mean ESCS quartile within country/economy	Reference slope: Q4 highest within-country school ESCS composition	-23.65	[-26.69, -20.14]	10
Mathematics	School mean ESCS quartile within country/economy	Q1 difference versus Q4 reference	13.19	[11.84, 16.30]	10
Mathematics	School mean ESCS quartile within country/economy	Q2 difference versus Q4 reference	2.29	[0.44, 4.95]	10
Mathematics	School mean ESCS quartile within country/economy	Q3 difference versus Q4 reference	-6.07	[-8.94, -1.15]	10
Creative thinking	Country peer-distraction burden quartile	Reference slope: Q4 highest country peer-distraction burden	-2.89	[-3.26, -2.49]	10
Creative thinking	Country peer-distraction burden quartile	Q1 difference versus Q4 reference	-1.04	[-1.32, -0.79]	10
Creative thinking	Country peer-distraction burden quartile	Q2 difference versus Q4 reference	-1.03	[-1.34, -0.60]	10
Creative thinking	Country peer-distraction burden quartile	Q3 difference versus Q4 reference	-1.83	[-2.77, -1.47]	10
Creative thinking	Country school phone-ban prevalence quartile	Reference slope: Q4 highest country phone-ban prevalence	-4.77	[-5.17, -4.23]	10
Creative thinking	Country school phone-ban prevalence quartile	Q1 difference versus Q4 reference	0.80	[0.07, 1.39]	10
Creative thinking	Country school phone-ban prevalence quartile	Q2 difference versus Q4 reference	1.47	[1.06, 2.07]	10
Creative thinking	Country school phone-ban prevalence quartile	Q3 difference versus Q4 reference	0.83	[0.29, 1.75]	10
Creative thinking	Student ESCS quartile within country/economy	Reference slope: Q4 highest within-country student ESCS	-3.23	[-3.74, -2.63]	10
Creative thinking	Student ESCS quartile within country/economy	Q1 difference versus Q4 reference	0.41	[-0.19, 0.85]	10
Creative thinking	Student ESCS quartile within country/economy	Q2 difference versus Q4 reference	0.64	[0.09, 1.35]	10
Creative thinking	Student ESCS quartile within country/economy	Q3 difference versus Q4 reference	-0.82	[-1.25, -0.11]	10
Creative thinking	School mean ESCS quartile within country/economy	Reference slope: Q4 highest within-country school ESCS composition	-1.55	[-2.09, -0.84]	10

Outcome	Context	Contrast	PV-mean B	PV range	PVs
Creative thinking	School mean ESCS quartile within country/economy	Q1 difference versus Q4 reference	-0.20	[-0.85, 0.48]	10
Creative thinking	School mean ESCS quartile within country/economy	Q2 difference versus Q4 reference	-1.86	[-2.38, -1.47]	10
Creative thinking	School mean ESCS quartile within country/economy	Q3 difference versus Q4 reference	-1.37	[-2.51, -0.79]	10

Notes:

Positive interaction estimates mean that the school peer-distraction slope is less negative than the Q4 reference; negative estimates mean a more negative slope than the Q4 reference.

Country peer-distraction burden quartiles were based on the country/economy weighted mean of individual perceived peer digital distraction. Q1: 20 systems, range 1.23-1.80; Q2: 20 systems, range 1.80-1.93; Q3: 20 systems, range 1.93-2.04; Q4: 20 systems, range 2.05-2.47.

Country school phone-ban prevalence quartiles were based on the country/economy weighted proportion of schools reporting that cell phones are not allowed on school premises. Q1: 20 systems, range 0.06-0.22; Q2: 20 systems, range 0.22-0.38; Q3: 20 systems, range 0.38-0.59; Q4: 20 systems, range 0.59-0.95.

Student and school ESCS quartiles were constructed within each country/economy. Full BRR-Fay inference can be produced by re-running run_brr_pv_contextual_stratification.py with replicate ranges and then pooling with pool_brr_pv_by_analysis.py.

Table S14. Demographic/SES-Adjusted Screening

This table reports final-student-weight, 10-plausible-value screening point estimates from a model adding ESCS, female student, immigrant background, and grade repetition to the full digital covariate model. It is intended to document robustness direction under additional demographic/SES controls. It is not a final BRR-Fay inferential table unless full replicate-weight verification is later completed.

Outcome	Term	PV-mean B	PV range	PVs
Mathematics	Leave-one-out school peer distraction	-27.93	[-29.47, -25.55]	10
Mathematics	Individual perceived peer distraction	-10.38	[-11.01, -9.39]	10
Mathematics	School peer distraction x phone ban	5.04	[2.92, 7.04]	10
Mathematics	Economic, social, and cultural status	22.91	[22.50, 23.41]	10
Mathematics	Female student	-10.78	[-11.68, -10.12]	10
Mathematics	Immigrant background	-5.37	[-6.50, -4.33]	10
Mathematics	Grade repetition	-49.68	[-50.63, -48.48]	10
Creative thinking	Leave-one-out school peer distraction	-2.72	[-3.06, -2.42]	10
Creative thinking	Individual perceived peer distraction	-1.26	[-1.39, -1.13]	10
Creative thinking	School peer distraction x phone ban	0.47	[0.02, 0.98]	10
Creative thinking	Economic, social, and cultural status	3.02	[2.97, 3.07]	10
Creative thinking	Female student	1.36	[1.19, 1.57]	10
Creative thinking	Immigrant background	-0.64	[-0.98, -0.33]	10
Creative thinking	Grade repetition	-6.87	[-7.15, -6.63]	10

Notes:

The school peer-distraction estimate remained negative in this demographic/SES-adjusted screening model for mathematics and creative thinking.

Full BRR-Fay verification can be produced with run_brr_pv_demographic_adjustment_sensitivity.py if the analysis is promoted to the formal supplement.

Demographic controls were coded as follows: female student from ST004D01T=1; immigrant background from IMMIG=2 or 3; grade repetition from REPEAT=1; ESCS from the PISA ESCS index.

Table S15. Secondary Life-Satisfaction Analysis

Outcome: PISA life satisfaction (ST016Q01NA, 0-10). Models used final student weights, 80 BRR-Fay replicate weights, country/economy fixed effects, and the same digital covariate set as the primary cognitive-outcome model.

Complete-case unweighted N = 67,748; weighted represented N = 2,915,653.

Term	B	SE	95% CI	p
School peer digital distraction, leave-one-out	0.129	0.082	[-0.032, 0.291]	0.117
Individual perceived peer distraction	-0.102	0.037	[-0.173, -0.030]	0.005
Own digital distraction in mathematics	-0.080	0.036	[-0.151, -0.009]	0.027
Digital device open in class	0.014	0.017	[-0.021, 0.048]	0.437
Leisure digital-resource use at school	0.032	0.018	[-0.003, 0.066]	0.073
Digital anxiety	-0.250	0.025	[-0.299, -0.202]	<.001
Pressure to be online/respond in class	-0.048	0.025	[-0.098, 0.001]	0.055
School cell-phone ban	0.141	0.192	[-0.236, 0.517]	0.465
School peer distraction x school cell-phone ban	-0.063	0.100	[-0.260, 0.133]	0.527

Note. Estimates are associational and should not be interpreted causally.
